# Pesticide Spraying May Spread Norovirus

**DOI:** 10.1289/ehp.121-a148

**Published:** 2013-05-01

**Authors:** Carol Potera

**Affiliations:** Carol Potera, based in Montana, has written for *EHP* since 1996. She also writes for *Microbe*, *Genetic Engineering News*, and the *American Journal of Nursing*.

Norovirus is the most common cause of viral foodborne illness worldwide.^1^ A new study suggests that contaminated water used to dilute or reconstitute agricultural pesticides may be one way the virus is entering the food supply.^2^

Farmers mix pesticides with water from sources including wells, irrigation ditches, rivers, and lakes. All these water sources have been known to harbor norovirus.^3^^,^^4^^,^^5^^,^^6^ Until recently, no one had tested whether norovirus in contaminated water remains infectious after pesticides are added. Now researchers at the National Institute for Public Health and the Environment in Bilthoven and the Institute for Risk Assessment Sciences at Utrecht University, the Netherlands, report that most pesticides do not counteract the infectivity of norovirus in contaminated water. Consequently, the water used to mix pesticides may be a microbial risk factor that could spread norovirus, conclude food technologist Katharina Verhaelen and her colleagues in the *International Journal of Food Microbiology*.^2^

The researchers tested four fungicides and four insecticides commonly used to protect fresh produce including lettuce and raspberries. These foods, especially raspberries, have been associated with several outbreaks of norovirus.^7^ The pesticides were diluted in sterile water to the highest concentrations recommended for crop spraying. In the laboratory, the pesticide solutions were spiked with one of two clinical strains of norovirus isolated from stools of infected people (hNoV GI.4 and hNoV GII.4), or with MNV-1, a mouse laboratory strain. When monitored for activity two hours later, all three norovirus types remained stable in seven of the eight tested pesticides.^2^

Generally in laboratory experiments, pesticides are neutralized to stop chemical activity at a certain time point—for example, by adding sodium thiosulfate to neutralize chlorine-based pesticides. However, the manufacturers of the commercial pesticides tested do not disclose their chemical composition, making it difficult to select appropriate neutralizing agents. Eliminating the neutralizing step also parallels what happens on farms; as Verhaelen points out, “In the practical application of pesticides the chemicals are not neutralized.”

The greatest microbial risk from pesticide-born norovirus would appear to be for produce such as soft berries like raspberries and strawberries, which are frequently sprayed shortly before harvest to reduce spoilage and then minimally processed before being eaten raw. “We’ve shown that noroviruses may persist during the shelf life of berries at different storage conditions,” Verhaelen says.^2^

**Figure:**
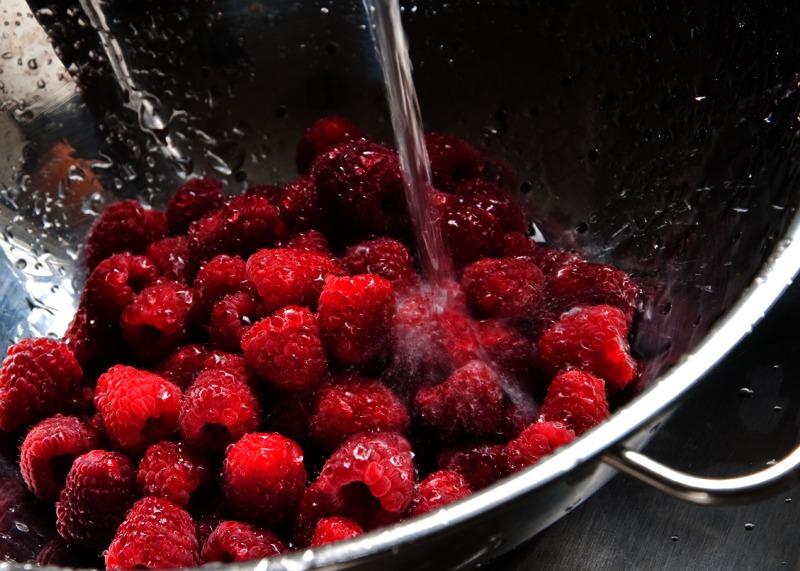
Fresh raspberries are one of the foods most commonly associated with outbreaks of norovirus. All produce should be washed before eating. © iStockphoto.com

Each year in the United States, norovirus causes 21 million cases of acute gastroenteritis, 70,000 hospitalizations, and 800 deaths. There are no drug treatments for norovirus infections and no vaccines to prevent them. Enduring a bout of norovirus does not leave a person immune to future infections.^8^

Verhaelen advises consumers to always wash produce. “The risk cannot be guaranteed to be reduced to zero, but the risk is lower compared to not washing,” she says.

Heat is a powerful way to reduce norovirus infectivity, and in one laboratory study heating raspberry puree spiked with MNV-1 to 65°C for 30 seconds reduced viral infectivity by about 99%.^9^ In another study, freezing did not destroy norovirus.^10^ In Finland, where several outbreaks of norovirus were traced to eating frozen raspberries, food safety experts recommend heating the frozen fruits for at least two minutes at 90°C before eating.^11^ However, heating fresh produce can negatively impact quality and nutritive value, notes Verhaelen.

Although cases of norovirus illness are epidemiologically linked to produce, “we do not have a full understanding of how norovirus enters the food chain,” says Kali Kniel, an associate professor of food parasitology and virology at the University of Delaware in Newark. She says the discovery that norovirus survives in pesticides is important, given water scarcities and the resulting increased reuse of water for application of pesticides and fertilizers.

## References

[1] Koo HL (2010). Noroviruses: the principal cause of foodborne disease worldwide.. Discov Med.

[2] Verhaelen K Persistence of human norovirus in reconstituted pesticides—pesticide application as a possible source of viruses in fresh produce chains. Int J Food Microbiol 160 3 323 328 2013); http//dx..org/. 10.1016/j.ijfoodmicro.2012.11.007 23290241

[3] Borchardt MA Incidence of enteric viruses in groundwater from household wells in Wisconsin. Appl Environ Microbiol 69 2 1172 1180 2003http//dx..org/. 10.1128/AEM.69.2.1172-1180.2003 12571044 PMC143602

[4] Cheong S Enteric viruses in raw vegetables and groundwater used for irrigation in South Korea. Appl Environ Microbiol 75 24 7745 7751 2009http//dx..org/. 10.1128/AEM.01629-09 19854919 PMC2794108

[5] Kishida N One-year weekly survey of noroviruses and enteric adenoviruses in the Tone River water in Tokyo metropolitan area, Japan. Water Res 46 9 2905 2910 2012http//dx..org/. 10.1016/j.watres.2012.03.010 22465727

[6] Horman A *Campylobacter* spp., *Giardia* spp., *Cryptosporidium* spp., noroviruses, and indicator organisms in surface water in southeastern Finland, 2000–2001. Appl Environ Microbiol 70 1 87 95 2004http//dx..org/. 10.1128/AEM.70.1.87-95.2004 14711629 PMC321284

[7] Hall AJ Epidemiology of foodborne norovirus outbreaks, United States, 2001–2008. Emerg Infect Dis 18 10 1566 1573 2012http//dx..org/. 10.3201/eid1810.120833 23017158 PMC3471645

[8] CDC. Norovirus Overview [website]. Atlanta, GA:U.S. Centers for Disease Control and Prevention (updated 12 Apr 2012). Available: http://goo.gl/3g69O [accessed 8 Apr 2013].

[9] Baert L The reduction of murine norovirus 1, *B. fragilis* HSP40 infecting phage B40-8 and *E. coli* after a mild thermal pasteurization process of raspberry puree. Food Microbiol 25 7 871 874 2008http//dx..org/. 10.1016/j.fm.2008.06.002 18721675

[10] Baert L (2008). Survival and transfer of murine norovirus 1, a surrogate for human noroviruses, during the production process of deep-frozen onions and spinach.. J Food Prot.

[11] Finnish Food Safety Authority Evira. Foreign Frozen Raspberries Are Recommended to Be Heated Before Eating [press release]. Helsinki, Finland:Finnish Food Safety Authority Evira (8 Jun 2009). Available: http://goo.gl/U0Jy7 [accessed 8 Apr 2013].

